# ELABELA/APELA Levels Are Not Decreased in the Maternal Circulation or Placenta among Women with Preeclampsia

**DOI:** 10.1016/j.ajpath.2018.04.008

**Published:** 2018-08

**Authors:** Natasha Pritchard, Tu'uhevaha J. Kaitu'u-Lino, Sungsam Gong, Justyna Dopierala, Gordon C.S. Smith, D. Stephen Charnock-Jones, Stephen Tong

**Affiliations:** ∗ Translational Obstetrics Group, Department of Obstetrics and Gynaecology, University of Melbourne, Heidelberg, Victoria, Australia; † Mercy Perinatal, Mercy Hospital for Women, Heidelberg, Victoria, Australia; ‡ Department of Obstetrics and Gynaecology, University of Cambridge; National Institute for Health Research Cambridge Comprehensive Biomedical Research Centre, Cambridge, United Kingdom; § Centre for Trophoblast Research, University of Cambridge, Cambridge, United Kingdom

## Abstract

The genetic deletion of apelin receptor early endogenous ligand (Elabela; official name *APELA*) produces a preeclampsia-like phenotype in mice. However, evidence linking ELABELA with human disease is lacking. Therefore, we measured placental mRNA and circulating ELABELA in human samples. ELABELA mRNA (measured by RNA sequencing) was unchanged in 82 preeclamptic placentas compared with 82 matched controls (mean difference, 0.53%; 95% CI, −25.9 to 27.0; *P* = 0.78). We measured circulating ELABELA in 32 women with preterm preeclampsia (delivered at <34 weeks' gestation) and 32 matched controls sampled at the same gestational age. There was no difference in circulating ELABELA concentrations in the preeclamptic cohort compared with controls (median, 28.5 pg/mL; 95% CI, 5.3 to 63.2 versus median, 20.5 pg/mL; 95% CI, 9.2 to 58.0, respectively); the median difference was 8.0 pg/mL (95% CI, −17.7 to 12.1; *P* = 0.43). In contrast, soluble *FLT1* (a protein with an established association with preeclampsia) mRNA was increased in placental tissue (mean difference, 34.9%; 95% CI, 16.6 to 53.1; *P* = 0.001), and circulating concentrations were 16.8-fold higher among the preeclamptic cohort (*P* < 0.0001). In conclusion, we were able to recapitulate the association between circulating soluble FLT1 and preeclampsia, but there was no association with ELABELA. The speculated clinical relevance of observations in the murine model linking ELABELA to preeclampsia likely are incorrect.

Preeclampsia is a major complication of pregnancy that is characterized by hypertension and can cause fetal growth restriction and injury to the maternal endothelium and multiple organs.^1^, ^2^

It is thought that inadequate remodeling of the maternal spiral arterioles by the extravillous trophoblast generates periodic placental malperfusion, leading to oxidative and endoplasmic reticulum stress.^1^, ^3^, ^4^ This causes increased placental release of anti-angiogenic factors such as soluble fms-like tyrosine kinase-1 (sFLT1)^5^ and soluble endoglin (ENG).^6^, ^7^ This leads to maternal endothelial dysfunction, which then causes hypertension and systemic organ injury.^8^

Apelin receptor early endogenous ligand (ELABELA; also known as ELA, official name is APELA) is a circulating peptide hormone that binds to the G-protein–coupled apelin receptor (APLNR).^9^ It has roles in embryonic stem cell renewal in mice^9^ and cardiac formation in zebrafish,^10^ but in adult humans its expression is restricted to the kidneys and the placenta.

Recently, Ho et al^11^ proposed decreased placental release of ELABELA as a causal factor in preeclampsia. Deletion of the murine *Elabela* gene caused preeclampsia-like symptoms with proteinuria, hypertension, and kidney injury during pregnancy. They proposed that loss of Elabela perturbs early placental vascular development, resulting in a placenta that is perfused inadequately. Ho et al^11^ showed that Elabela may have paracrine roles in placental development, acting on fetal endothelial cells to facilitate normal branching angiogenesis and formation of the labyrinth network, allowing proper placental exchange.

As an additional mechanism, decreased circulating ELABELA was postulated to contribute to the maternal systemic injury seen in preeclampsia. Circulating ELABELA may act directly on maternal endothelial cells promoting vasodilation^12^ and regulating diuresis and fluid balance.^9^, ^13^, ^14^, ^15^ In mice, Elabela protects against acute kidney injury,^16^ an organ commonly injured in preeclampsia, and reduces blood pressure in the setting of pulmonary hypertension.^17^ In support of this, Ho et al^11^ showed that circulating Elabela levels correlated with the severity of maternal proteinuria and kidney damage in mice. Notably, administering Elabela to pregnant *Elabela* null mice from gestational day 7.5 prevented the increase in maternal blood pressure observed at gestational day 16 in *Elabela*-null mice. This treatment also prevented fetal weight loss and proteinuria with no adverse effects on the fetus.

Although these studies strongly support the hypothesis that ELABELA is implicated in preeclampsia, as yet there is no evidence that ELABELA levels are reduced in human disease. Of the two postulated mechanisms, there is no obvious way to confirm that ELABELA is indeed deficient in early placental development among those destined to develop preeclampsia. However, it is possible to measure ELABELA mRNA in the delivered placenta and to determine whether circulating ELABELA is deficient among patients with preeclampsia. Therefore, we measured placental ELABELA mRNA and circulating ELABELA in samples obtained from pregnant women with preeclampsia and healthy controls.

## Materials and Methods

The Pregnancy Outcome Prediction study was a prospective cohort of 4512 nulliparous pregnant women with a viable singleton pregnancy attending the Rosie Hospital (Cambridge, UK) between January 14, 2008, and July 31, 2012. This study has been described previously in detail.^18^, ^19^ Preeclampsia was defined (for all participants in this report) according to the 2013 American College of Obstetricians and Gynecologists criteria^20^ (Table 1).

**Table 1:** ELABELA/APLN/APLNR/FLT1 Placental Samples: Clinical Characteristics

Patient characteristic	Pregnancy controls (*n* = 82)	Preeclampsia (*n* = 82)	*P* value
Maternal age at delivery (years), median (IQR)	30.2 (25.6–32.7)	29.6 (25.9–33.4)	0.545
Gestation at delivery (weeks), median (IQR)	40.4 (39.4–40.9)	40.1 (38.7–40.8)	0.01
BMI (kg/m^2^), median (IQR)	26.3 (23.7–30.0)	26.7 (23.6–31.1)	0.002
Highest SBP before labor (mmHg), median (IQR)	120 (116–130)	140 (129.8–150)	<0.0001
Highest DBP before labor (mmHg), median (IQR)	75.5 (70–80)	90.0 (80–98)	<0.0001
Birthweight (g), median (IQR)	3550 (3380–3759)	3530 (3083–3810)	0.062
Smoking, *n*	10	11	1

All women were nulliparous. Cases and controls were perfectly matched for fetal sex, labor status, and caesarean section. The Wilcoxon rank-sum test was used to compare medians. The McNemar test was used to compare paired nominal data (discordant smoking status, 3 pairs out of 82).

APLN, apelin; APLNR, apelin receptor; BMI, body mass index; DBP, diastolic blood pressure; ELABELA, apelin receptor early endogenous ligand; IQR, interquartile range; SBP, systolic blood pressure; FLT1, Fms-like tyrosine kinase-1.

Placental tissue was obtained from women with preeclampsia (*n* = 82) and from healthy matched controls (*n* = 82). Controls were matched for laboring status, fetal sex, cesarean section, smoking, maternal body mass index, gestational age, and maternal age. All participants provided written informed consent and ethical approval for the study was given by the Cambridgeshire 2 Research Ethics Committee (07/H0308/163).

Plasma samples were obtained from women with preterm preeclampsia delivering at a tertiary hospital in Melbourne, Australia, between June 2012 and October 2017. Blood was obtained from women with preterm preeclampsia (*n* = 32) and healthy pregnancies (*n* = 32) who delivered without complications at full term, and were matched for maternal age and parity. Ethical approval was obtained from The Mercy Health Human Research Ethics Committee (R11/34). Preterm preeclampsia was defined as preeclampsia necessitating delivery at <34 weeks' gestation for maternal or fetal indications (Table 2).

**Table 2:** ELABELA/sFlt1/Soluble ENG Plasma Samples: Clinical Characteristics

Patient characteristic	Pregnancy controls (*n* = 32)	Preeclampsia (*n* = 32)	*P* value
Maternal age at delivery (years), median (IQR)	32.4 (29.2–35.3)	32.7 (29.7–35.6)	0.518
Gestation at delivery (weeks), median (IQR)	39.6 (38.7–40.5)	29.7 (27.8–31.5)	<0.0001
Gestation at blood collection (weeks), median (IQR)∗	28.4 (26.7–30.4)	29.4 (27.4–30.9)	0.254
BMI (kg/m^2^), median (IQR)†	25 (22–29)	28.5 (25–34)	0.008
Parity, n (%)			
0	15 (46.9)	22 (68.8)	0.208
1	12 (37.5)	7 (21.9)	
≥2	5 (15.6)	3 (9.4)	
Highest SBP during admission (mmHg), median (IQR)	125 (120–130)	170 (160–180)	<0.0001
Highest DBP during admission (mmHg), median (IQR)	78 (72–82)	100 (91–109)	<0.0001
Birthweight (g), median (IQR)	3425 (3158–3668)	1078 (810–1403)	<0.0001

The *U*-test was used to compare medians and the χ^2^ test was used to compare categoric variables.

BMI, body mass index; DBP, diastolic blood pressure; ELABELA, apelin receptor early endogenous ligand; ENG, endoglin; IQR, interquartile range; SBP, systolic blood pressure; sFLT1, soluble fms-like tyrosine kinase-1.

∗ Gestation at blood collection was available for 31 of 32 controls.

† BMI data were available for 31 of 32 controls and 25 of 32 preeclamptic patients.

Placental villous samples were collected within 30 minutes of delivery and processed as previously described.^19^ Total RNA was extracted using the mirVana miRNA Isolation Kit (Thermo Fisher Scientific, Waltham MA) followed by DNase treatment (DNA-free DNA Removal Kit; Thermo Fisher Scientific). Sequencing libraries were prepared from 300 to 500 ng of total RNA with the TruSeq Stranded Total RNA Library Prep Kit with Ribo-Zero Human/Mouse/Rat (Illumina, San Diego, CA). Libraries were quantified and sequenced using Illumina HiSeq2500 and HiSeq4000 instruments. To analyze the RNA sequencing data we used Bioconductor, cutadapt, Tophat2, subbread, ENSEMBL and R. Primer sequences and poor-quality bases were trimmed using cutadapt version 1.8.1^21^ and the reads were mapped to the human genome reference (GRCh38) using a two-pass alignment protocol with TopHat 22.0.12.^22^ Uniquely mapped reads were quantified with Subread/featureCounts version 1.5.1 (*http://subread.sourceforge.net*) using the ENSEMBL version 82 (*https://www.ensembl.org*) transcriptome definitions. Transcript abundance was measured in fragments per million mapped reads using the Bioconductor package DESeq2 version 3.0 (*http://bioconductor.org*). The mRNAs encoding ELABELA, apelin, APLNR, FLT1, and ENG were examined. The statistical significance of the fold-changes were tested by the Wilcoxon signed-rank test in R version 3.1.1 (*https://cran.r-project.org*).

The following analytes were measured in plasma: ELABELA (human enzyme immunoassay, manufactured by Peninsula Laboratories, Inc., cat S1508.0001; supplied by Resolving Images, Preston, Australia); sFLT1 (DuoSet VEGF R1/FLT1; R&D Systems Bioscience, Waterloo, Australia), and soluble ENG (DuoSet Human Endoglin; R&D Systems, Waterloo, NSW, Australia). All assays were performed according to the manufacturers' instructions without further validation.

Continuous variables were assessed using the *U*-test for unpaired nonparametric data, and the Wilcoxon signed-rank test for paired nonparametric data. Receiver operated characteristic curves were generated. Independence between categoric variables was assessed with the χ^2^ test and the McNemar test for paired nominal data (smoking status). Statistical analysis was conducted using GraphPad Prism 7 (GraphPad Software, La Jolla, CA) and in R. *P* < 0.05 was considered significant.

## Results

**Figure 1:**
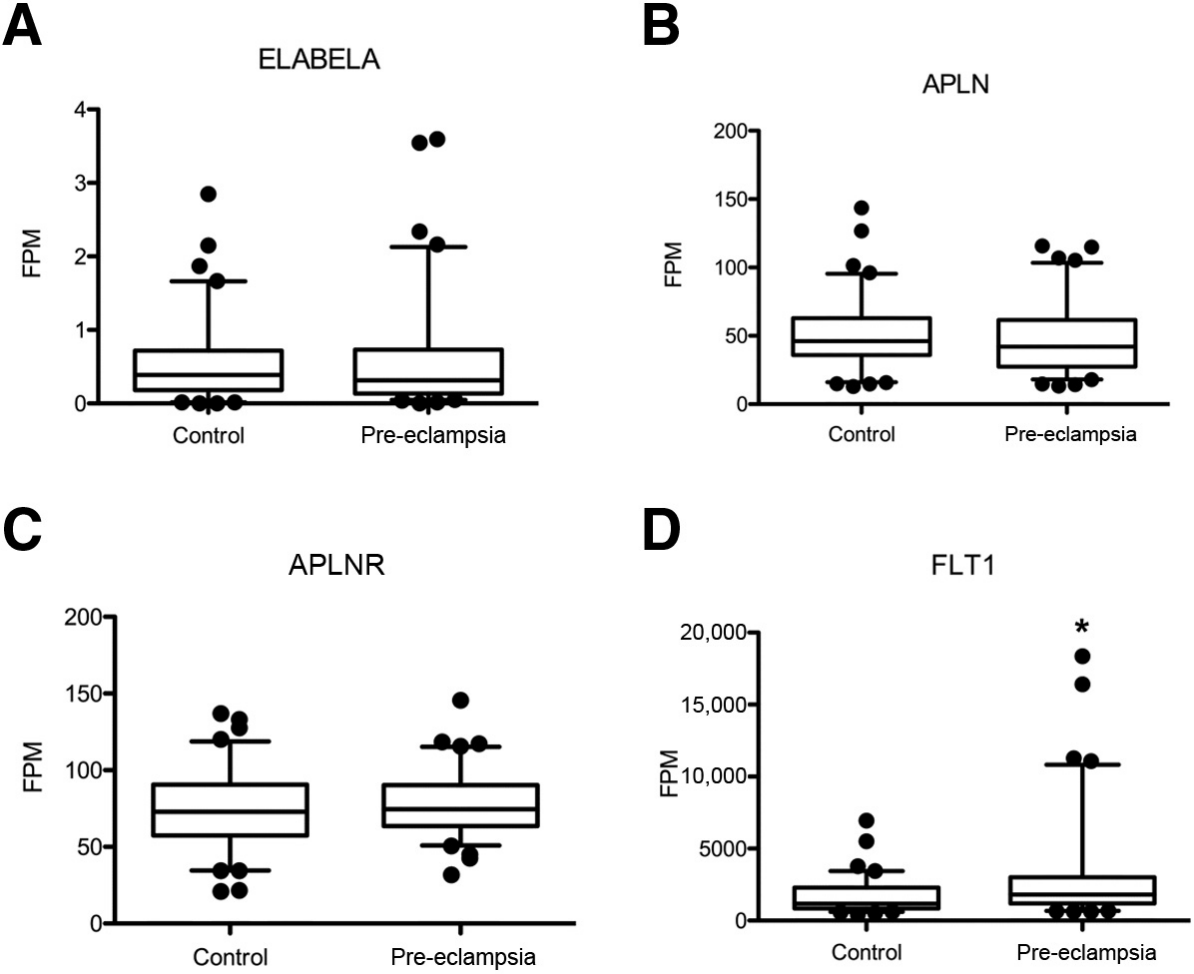
Placental apelin receptor early endogenous ligand (ELABELA) mRNA is not changed in preeclampsia. **A–C:** ELABELA (**A**), apelin (*APLN*; **B**), and apelin receptor (*APLNR*; **C**) mRNA were not altered significantly between groups. **D:** Fms-like tyrosine kinase-1 (*FLT1*) mRNA was altered significantly between groups. In all cases, the Wilcoxon signed-rank test was used and data are shown with **boxes** representing the median ± interquartile range and **bars** representing the median ± 5th and 95th centile. mRNAs were measured in preeclamptic (*n* = 82) and control (*n* = 82) placentas. ^∗^*P* < 0.001 versus control. FPM, fragments per million.

The levels of mRNA in the placentas of 82 women with preeclampsia were examined at term and in healthy matched controls. High-quality RNA (RNA integrity number, 7.0 to 9.5; median, 8.2) was purified and approximately 100 million 125-base single-end sequencing reads were collected for each sample. The abundance of the mRNAs of interests was determined. There were no differences in the placental mRNAs encoding ELABELA [mean fragments per million in control and pre-eclampsia, respectively, were as follows: 0.532 (95% CI, 0.417 to 0.648) and 0.567 (95% CI, 0.415 to 0.719; *P* = 0.78), apelin, 50.5 (95% CI, 45.1 to 55.8) and 47.8 (95% CI, 42.4 to 53.2; *P* = 0.448), nor APLNR, the receptor for these ligands, 74.5 (95% CI, 69.2 to 79.9) and 77.8 (95% CI, 73.5 to 82.1; *P* = 0.398)] (Figure 1, A–C). In contrast, FLT1 mRNA levels (sFLT1 is a splice variant of FLT1, the increase of which is associated consistently with preeclampsia) were 1.87-fold higher in women with preeclampsia (*P* = 0.001) (Figure 1D). ENG mRNA was 1.28-fold higher (ENG, *P* = 0.001, data not shown).

We then measured circulating ELABELA concentrations among 32 women with early onset preeclampsia (delivered <34 week's gestation) and compared these with levels in plasma samples collected from 32 healthy controls (Table 2). Although gestational age at delivery was significantly different between the two groups (39.6 versus 29.7 weeks' gestation; *P* < 0.001), gestation at the time of blood collection was equivalent (28.4 and 29.4 weeks' gestation). There were no differences between the controls in maternal age or parity. Body mass index and blood pressure were higher in the preeclampsia cohort.

**Figure 2:**
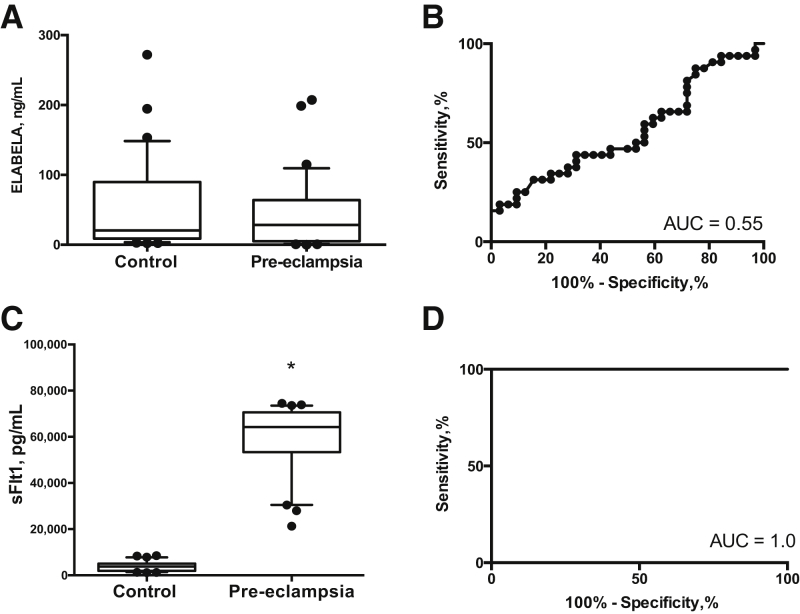
Circulating apelin receptor early endogenous ligand (ELABELA) is not decreased in preeclampsia. **A** and **B:** ELABELA levels were not altered significantly between groups: *U*-test (**A**) with the receiver operator curve (**B**) producing an area under the curve (AUC) of 0.55. **C** and **D:** In contrast, circulating soluble fms-like tyrosine kinase-1 (sFLT1) levels were significantly higher when measured in the preeclamptic cohort (**C**), with a receiver operator area under the curve of 1.0 (**D**). Data are shown with **boxes** representing the median ± interquartile range and **bars** representing the 5th to 95th percentile. Plasma levels of proteins detected by human enzyme immunoassay in preeclamptic women (*n* = 32) and controls (*n* = 32) matched for gestation at blood sampling. ^∗^*P* < 0.0001 versus control.

There were no differences in circulating ELABELA levels in the preeclamptic cohort compared with controls (preeclamptic cohort: median, 28.5 pg/mL; 95% CI, 5.3 to 63.2 versus controls: median, 20.5 pg/mL; 95% CI, 9.2 to 58.0; median difference, 8.0 pg/mL; 95% CI, -17.7 to 12.1; *P* = 0.433) (Figure 2, A and B). In contrast, plasma sFLT1 was 16.8-fold higher in preeclamptic women (*P* < 0.0001) (Figure 2C). There was perfect discrimination between the groups (Figure 2D). Soluble endoglin levels also were significantly higher in the women with preeclampsia (*P* = 0.002; area under the curve, 0.77, not shown).

## Discussion

The demonstration that pregnant *Elabela*-null mice developed preeclampsia-like symptoms led Ho et al^11^ to propose a role for reduced placental ELABELA production and release in the pathophysiology of preeclampsia. However, they did not show ELABELA was reduced in human disease. No evidence of any differences was found in ELABELA placental transcript abundance or circulating protein levels among cases of preeclampsia, compared with controls.

In *Elabela*^-/-^ mice major defects in the placental labyrinth were evident by embryonic day 10.5 and more significantly at embryonic day 18.5. At this time, placental *sFlt1* mRNA also was increased in the null mice. However, in human placenta ELABELA mRNA did not differ between patients with preeclampsia and healthy controls even though *sFLT1* mRNA was increased. Furthermore, the mRNAs encoding other components of the apelin-APLNR signaling axis (apelin and APLNR) did not differ in the placentas of women with preeclampsia. This suggests that gross alterations of this system are not associated with preeclampsia in the placenta at gestation when the disease becomes clinically evident, although the possibility remains that ELABELA deficiency might impair early fetal endothelial and placental development. In contrast to our negative findings with ELABELA, we observed strong associations between sFLT1 and preeclampsia in women. Hence, the negative findings in relation to ELABELA are unlikely to be owing to methodologic flaws in the assays used or the classification of cases. Our findings do not support the hypothesis that ELABELA perturbation plays a major role in preeclampsia in women.

## Supplemental Data

Data Profile
